# Whole-genome sequencing and characterization of *Pseudomonas stutzeri* P1 endophyte isolated from potato unveils plant growth-promoting and other traits

**DOI:** 10.3389/fmicb.2026.1848650

**Published:** 2026-08-21

**Authors:** Poonam Patel, Krutarth Raval, Satyamitra Shekh, Payal Mavadiya, Snehal Bagatharia, Fenil Patel, Amrutlal Patel

**Affiliations:** Gujarat Biotechnology Research Center (GBRC), Gandhinagar, Gujarat, India

**Keywords:** genome annotation, PGPT-associated genes, plant endophyte, plant growth promoting traits (PGPTs), potato (*Solanum tuberosum* L.), *Pseudomonas stutzeri*

## Abstract

Endophytic bacteria play an important role in plant growth promotion and stress tolerance, offering sustainable alternatives to chemical inputs in agriculture. In this study, an endophytic bacterial strain P1 was isolated and identified as *Pseudomonas stutzeri*, a plant-associated bacterium exhibiting multiple plant growth–promoting traits (PGPTs). Biochemical (qualitative and quantitative) and *in vitro* analyses demonstrated nitrogen fixation, phosphate solubilization, ammonia production, indole-3-acetic acid (IAA) production, biofilm formation, and tolerance to abiotic stresses, including salinity and drought. Furthermore, the P1 strain displayed strong biocontrol activity against the fungal pathogen *Fusarium oxysporum* f. sp. *cumini,* indicating its potential to mitigate biotic stress. Whole-genome sequencing generated a high-quality complete genome of 4,758,235 bp. Functional annotation showed enrichment of metabolic pathways associated with plant-microbe interactions and environmental adaptation. Further analyses using KEGG and PGPT-pred data confirmed the presence of genes associated with direct and indirect PGPT, such as nitrogen fixation, phosphate solubilization, biofilm formation, and stress tolerance. The genome also contained genes related to CAZymes, adhesion, and motility, highlighting a strong plant association, whereas the genome lacked major virulence factors and antimicrobial traits, supporting the non-pathogenic nature of the P1 strain. Overall, these findings demonstrate the potential of P1 as a promising bioinoculant candidate for sustainable agriculture in the potato sector.

## 1 Introduction

Potato (*Solanum tuberosum* L.) is one of the most widely consumed tubers worldwide due to its high nutritional value and broad adaptability across diverse agro-climatic zones (Hu et al., 2025). India ranks as the world’s second-largest potato producer, with an average production of 59.5 million metric tonnes in 2024–2025 (Market Intelligence Cell (MIC) for APEDA, 2025). For the cultivation of potato plants, high fertilizer inputs are required for optimum productivity, which can be achieved through high-yielding varieties (Asakaviciute et al., 2025), such as Kufri Ratan, Kufri Chipsona, Kufri Tejas, and Kufri Chipbharat-1 (Anonymous, 2025), along with enhanced micropropagation strategies (Patel et al., 2025b). Additionally, biotization with endophytic bacteria has emerged as an effective approach for promoting plant growth, where bacterial genera such as *Bacillus, Azospirillum,* and *Bradyrhizobium* have been demonstrated to promote plant survival and increase potato yields (Molina et al., 2025).

The association between the plant and the endophytic bacteria improves plant health and tolerance to various biotic and abiotic stresses (Kanani et al., 2020). The endophytic bacteria often contribute in terms of phosphate solubilization, nitrogen fixation, siderophore secretion, secondary metabolite production, phytohormone production such as auxins and cytokinins, tolerance to drought/salt, and various other biocontrol activities against pathogens (Patel et al., 2025a), altogether known as Plant Growth Promoting Traits (PGPTs). Molina et al. (2025) reported *Azospirillum brasilense* Ab-V5 and *Rhizobium tropici* CIAT 899 as potent bacterial inoculants that promoted plant growth and enhanced tuber formation. *Bacillus halotorens* Q2H2 showed strong antagonistic effects against pathogenic *Fusarium* sp. and promoted plant growth (Wang et al., 2024). Likewise, *Bacillus velezensis* exhibited positive attributes in terms of IAA production, siderophore production, nitrogen fixation, phosphate solubilization, and overall plant growth (Shuang et al., 2022). Moreover, the strain IMBG294 of *Pseudomonas fluorescens* also showed biocontrol activity against soft rot disease (*Pectobacterium atrosepticum*) in potato (Pavlo et al., 2011).

The recent advances in next-generation sequencing (NGS) technologies have enhanced the in-depth understanding of microbial genomes from a functional and genomic viewpoint (Kaul et al., 2016). The comprehensive genome analysis of endophytic bacteria isolated from plants unravels various metabolic pathways, underlying cross-talks and complex networks, thereby providing valuable insights and directions for future research (Satam et al., 2023).

Although several *Pseudomonas* sp. with PGPT have been widely reported, genomic-level studies on *Pseudomonas stutzeri*, particularly trait analyses associated with potato, remain unexplored. The study addresses this knowledge gap in detail by investigating genomic features, biochemical traits associated with plant growth promotion, and genome-level elucidation from the perspective of plant growth promotion in potato. The findings from the study may help to generate crop-specific bioinoculants to improve agricultural yield and biocontrol efforts, particularly in potatoes.

## 2 Materials and methods

### 2.1 Isolation of an endophytic bacterial isolate (P1)

An endophytic bacterium designated P1 was isolated from the nodal segment of an *in vitro*-raised potato plant (Jasim et al., 2014). The bacterial culture was streaked onto a Nutrient Agar (NA) plate (Himedia, India) and incubated at 37 °C for 24 h to ensure growth under laboratory conditions. A single colony corresponding to isolate P1 was purified by repeated streaking onto NA plates. For long-term storage, the endophytic bacterial culture was maintained at −80 °C in a 15% glycerol stock.

### 2.2 Identification of endophytic bacteria using MALDI-TOF MS

The identification of endophytic bacteria was performed using Matrix-Assisted Laser Desorption Ionization Time-of-Flight Mass-Spectroscopy (MALDI-TOF MS) (Toubal et al., 2018). The single colony of endophytic bacteria was directly smeared (a thin film) as a spot on the MALDI target plate; the spot was overlaid with 70% (v/v) formic acid and air dried at room temperature. Again, the spot was covered with 1 μL of *α*-cyano-4-hydroxycinnamic acid (CHCA) in 50% (v/v) acetonitrile and left to dry at room temperature. The resultant spectra were compared with reference spectra using Biotyper 3.1 (Bruker MALDI Biotyper, UK). The classification was carried out using the direct transfer method (Autoflex® maX, Bruker MALDI User’s Booklet, Bruker Daltonics Inc.). According to manufacturer guidelines, scores ≥ 2.0 indicate high-confidence species identification, whereas scores 1.70–1.99 show low confidence, and scores 0.00–1.69 show no reliable identification.

### 2.3 PGPT evaluation based on biochemical assay

#### 2.3.1 Biological nitrogen-fixing ability

The nitrogen-fixing ability of the bacteria was assessed using Ashby’s nitrogen-free agar medium. The bacterial strain was streaked on Ashby nitrogen-free agar medium and incubated at 37 °C, and the plates were observed for bacterial growth for 7 days (Kizilkaya, 2008).

#### 2.3.2 Phosphate solubilization

The phosphate solubilization capacity of the bacteria was determined by using Pikovskaya’s agar medium (Himedia, Nashik, India). After streaking the bacteria on Pikovskaya’s agar medium, the plates were incubated at 37 °C for 7 days. The transparent halo zone around bacterial growth was recorded (Nautiyal, 1999).

Further, phosphate solubilization was estimated quantitatively as described by Prasad et al. (2022) on Botanical Research Institute’s phosphate (NBRIP) growth media (10 g glucose, 5 g MgCl_2_.6H_2_O, 5 g Ca_3_(PO_4_)_2_, 0.25 g MgSO_2_.7H_2_O, 0.2 g KCl, 0.1 g (NH_4_)_2_SO_4_, 1.5% agar to the volume of 1 L, and pH adjusted to 7.0) (Batista et al., 2021). Initially, the culture was grown in 10 mL LB broth overnight to get an active culture, then centrifuged at 6,000 rpm for 10 min. The pellet was washed with phosphate-free NBRIP medium. The cells were resuspended and adjusted to an optical density (OD600) of 0.1. Subsequently, 50 mL of NBRIP medium was inoculated with the standardized cell suspension. Uninoculated NBRIP medium was used as the control. The culture was incubated at 30 °C for 5 days. Culture supernatant (500 μL), 10% (w/v) trichloroacetic acid (500 μL), and 4 mL of a color reagent, composed of a 1:1:1:2 ratio of 3 M H_2_SO_4_, 2.5% (w/v) ammonium molybdate, 10% (w/v) ascorbic acid, and distilled water, were well mixed and incubated at room temperature (26 ± 2 °C) for 15 min. The absorbance of the gradually developing blue color was quantified at 820 nm utilizing a spectrophotometer in the Cytation 5 (Agilent BioTek Instruments, Winooski, VT, USA). The solubilized phosphate concentration was determined using a standard curve prepared (0, 20, 40, 60, 80, 100 μg mL^−1^) with potassium dihydrogen phosphate (KH_2_PO_4_).

#### 2.3.3 Amylase production

To assess amylase production, the bacterial strain P1 was streaked onto Starch Casein Agar (SCA) media and incubated at 37 °C for 7 days. The amylase activity was checked by flooding the plate with Gram’s Iodine. The halo zone around the bacterial colonies represents starch hydrolysis (Sanjaya et al., 2024).

For quantitative analysis, the liquid Yeast Phosphate Soluble Starch (YpSs) medium was used to produce crude amylase enzyme. *Pseudomonas stutzeri* P1 was inoculated into YpSs broth and incubated at room temperature for 24 h until the culture reached an OD of 0.5. Then the culture was centrifuged at 5,000 rpm for 5 min at 4 °C, and the supernatant was collected as the crude enzyme. Amylase activity of *Pseudomonas stutzeri* P1 was determined using the DNS (3,5-dinitrosalicylic acid) method on days 1 and 5 with slight modifications (Miller, 1959; Nisa et al., 2021). Briefly, 1 mL of crude enzyme was mixed with 1 mL of substrate (1% soluble starch) and incubated at 40 °C for 5 min. The reaction was stopped by adding 1 mL of DNS reagent, followed by boiling for 5 min. Absorbance was measured at 540 nm using a spectrophotometer. A glucose standard curve was prepared to determine the concentration of reducing sugars released during the reaction with the variable concentrations (0, 100, 200, 300, 400, 500, 600 mg L^−1^). Amylase activity was calculated from the glucose concentration using the following formula described by Kombong (2004).


$$\begin{array}{l} \text{Amylase Activity}\;(\frac{U}{\mathrm{mL}})\\ =\frac{\left(\text{Glucose released}\times \text{Total volume of enzyme substrate mixture}\right)}{\left(\text{Molecular weight of glucose}\times \text{Incubation time}\times \text{Enzyme volume}\right)} \end{array}$$

#### 2.3.4 Siderophore activity

The siderophore activity of the bacteria was determined based on the formation of a halo zone around colonies on Chrome Azurol S (CAS) medium supplemented with ferric chloride (FeCl_3_) as the iron source, incubated at 37 °C for 7 days (Himpsl and Mobley, 2019).

#### 2.3.5 Catalase activity

To assess catalase activity, the bacterial colony was smeared on a glass slide, and 1–2 drops of 3% hydrogen peroxide (H_2_O_2_) were added. The presence and absence of bubble formation were recorded (Taylor and Achanzar, 1972).

#### 2.3.6 Ammonia production

The nitrogen-fixing ability of bacteria to produce ammonia was quantified using the Nessler method with minor modification (Kifle and Laing, 2016). The freshly grown bacterial culture was inoculated into 10 mL of peptone water and incubated for 72 h at room temperature with 172 rpm rotation. After incubation, the culture was centrifuged at 10,000 rpm for 5 min, and 2.5 mL of supernatant was collected and diluted with 2.5 mL of distilled water; 0.5 mL of Nessler’s reagent was then added. Samples were incubated for 10 min to observe the color change from yellow to orange, indicating that bacteria produce ammonia, which was measured at 425 nm. A calibration curve was prepared using NH_4_Cl as a standard at concentrations of 0, 20, 60, 80, and 100 μg mL^−1^ of NH_3_. Notably, Nessler’s reagent primarily detects ammonium ions but also reacts with other nitrogen-containing compounds. Therefore, the values represent the total concentration of ammonia and other reactive nitrogenous compounds as well as those produced by bacterial cells (Prasgi and Cahyani, 2025).

#### 2.3.7 IAA production test

IAA production by the bacterial isolate was determined using a colorimetric assay with Salkowski reagent (50 mL of 35% perchloric acid and 1 mL of 0.5 M FeCl_3_), as described by Marakana et al. (2018). The bacteria were inoculated on Luria–Bertani (LB) broth supplemented with 1% tryptophan and incubated at 37 °C for up to 7 days. Aliquots of bacterial culture (1 mL) were collected periodically on the 1st, 2nd, 4th, and 6th days of incubation and mixed with 2 mL of Salkowski reagent (1:2 ratio). The reaction mixture was incubated in the dark for 1 h, and IAA production was determined by measuring absorbance at 530 nm.

#### 2.3.8 Biofilm production

The biofilm formation assay was performed using crystal violet dye with minor modifications, as previously described by Budil and Lišková (2024). The test was performed on a 96-well polystyrene plate. The freshly grown culture (10 μL) was inoculated into 1/10th LB broth, 1/10th LB broth with 0.5% glucose, and 1/10th LB broth with 1% glucose. The plates were incubated at 37 °C for 48 h. After incubation, the OD was taken at 600 nm. Furthermore, the media was drained, and a 0.1% crystal violet solution (200 μL) was added to the wells, which were then incubated for 30 min.

Three washes were given with 1X PBS buffer; ethanol was added, and the OD was taken at 570 nm. The ability of bacteria to form biofilm was quantified as the biofilm formation index (BFI), calculated as follows: [OD 570 nm (P1) – OD 570 nm (Control)]/OD 600 nm (cells) (Lin et al., 2020). The isolate was categorized as follows: strong, moderate, weak, or non-biofilm producers based on the classification (OD ≤ ODc: none; ODc – 2 × ODc: weak; 2–4 × ODc: moderate; > 4 × ODc: strong) as described by Hu et al. (2016).

### 2.4 Abiotic and biotic stress tolerance assay

#### 2.4.1 Salt tolerance assay

The salt stress tolerance of strain P1 was assessed by inoculating the bacteria in LB broth with various concentrations of sodium chloride (NaCl) at 0, 5, 10, 15, and 20%. The tubes were incubated for 7 d at 37 °C, and the turbidity of bacterial cells was measured at OD 600 nm. The sterile medium was used as a blank (Singh et al., 2015).

#### 2.4.2 Drought tolerance assay

The efficacy of drought tolerance was assessed by incubating the P1 strain under drought stress conditions. Drought stress was induced by adding polyethylene glycol (PEG 4000) at various concentrations of 0, 5, 10, 15, and 20% in LB broth. The P1 isolate was inoculated in LB broth, incubated for 7 d at 37 °C, and the turbidity of bacterial cells was measured at OD 600 nm. The sterile medium was used as a blank. The isolate was categorized as > 0.5: completely tolerant; 0.4–0.5: tolerant; 0.3–0.39: sensitive; and OD < 0.3: highly sensitive, as described by Ashry et al. (2022).

#### 2.4.3 Antifungal activity

The antifungal activity of bacterial isolate P1 was assessed against the phytopathogenic fungi by using the dual-culture assay method (Soliman et al., 2022). For this, *Fusarium oxysporum* f. sp. *cumini* and *Aspergillus niger* were selected because they cause significant damage to potato crops (Mamaghani et al., 2024). A 5 mm fungal mycelial disc of each fungus was placed on one side of the plate containing a 1:1 mixture of NA and Potato Dextrose Agar (PDA), while bacterium P1 was streaked on the opposite side of the plate. The control plates containing the respective fungal cultures were maintained separately. All plates were incubated at 28 °C for 7 days. The biocontrol activity was evaluated based on the reduction of fungal growth compared to the growth on the respective control plate (Kumar et al., 2022). The percentage inhibition of fungal growth was calculated using the formula: Percentage inhibition (%) = [(C − T)/C] × 100, where C represents fungal growth in the control plate, and T represents fungal growth in the treatment plate (Soliman et al., 2022).

### 2.5 Whole-genome sequencing and data analysis

#### 2.5.1 DNA extraction, library preparation and genome assembly

**Figure 1:**
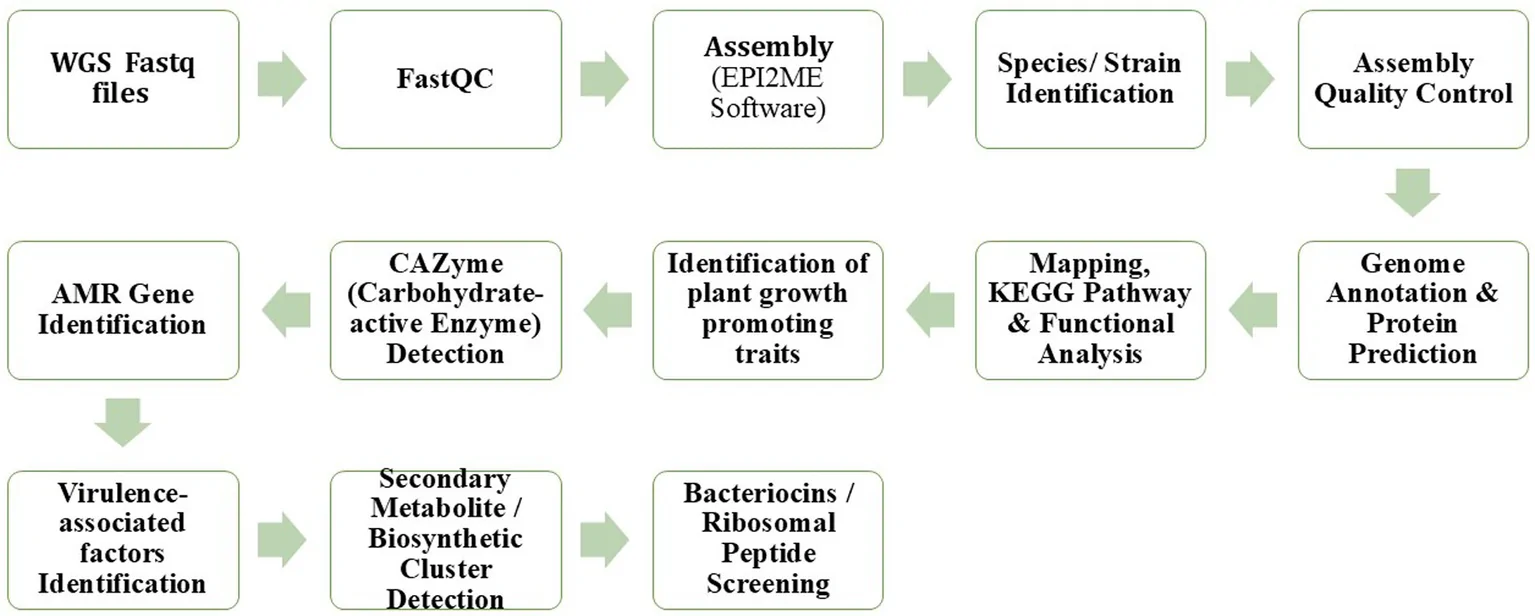
Whole-genome sequencing pipeline for plant endophyte P1 strain isolated from an *in vitro*-raised potato plant.

Genomic DNA was extracted using the QIAamp® DNA Micro Kit (QIAamp Mini, Germany) following the protocol as described by the manufacturer. The genomic libraries were prepared using the Ligation Sequencing gDNA-Native Barcoding Kit 96 V14 (SQK-NBD114.96) (Oxford Nanopore Technologies (ONT), Oxford, UK) as per the manufacturer’s protocol. The libraries were then sequenced using the Oxford Nanopore GridION sequencer (ONT). After sequencing, raw reads were processed using the automated EPI2ME software (Oxford Nanopore Technologies, UK) via a bacterial assembly and annotation workflow with *de novo* assembly for mapping. The quality of raw Nanopore reads was further evaluated with FastQC, while circular genome visualization and genomic architecture analysis were performed using Proksee.^1^ For further analysis, the pipeline was followed as outlined in Figure 1.

#### 2.5.2 Genome-based taxonomic profiling and annotation

The assembled genome was further analyzed using multiple tools for genome identification and taxonomic profiling. TYGS was used for phylogenetic identification based on comparisons of whole-genome and digital DNA–DNA hybridization (dDDH) values (Meier-Kolthoff and Göker, 2019). PubMLST uses housekeeping gene allelic profiles for bacterial identification (Matsui et al., 2018). Barrnap v.0.9 uses prediction based on the ribosomal RNA gene (16S rRNA) for taxonomic confirmation (Seemann, 2014), and DFAST was used for automated structural and functional genome annotation (Tanizawa et al., 2018). For assembly quality assessment, QUAST v5.2.0 (Gurevich et al., 2013) and BUSCO v5.7.1 software were used to evaluate genome completeness and contamination (Simão et al., 2015). Genome annotation and protein prediction were performed using Prokka (Seemann, 2014). Functional annotations were predicted using the eggNOG-mapper v2 web server with an E-value threshold < 0.001, identifying core genes, accessory genes, and singletons (Cantalapiedra et al., 2021). Gene enrichment was conducted using ShinyGO v0.85 (Ge et al., 2020), whereas KEGG pathway mapping was performed using Blast KOALA to identify metabolic and functional pathways in the P1 genome (Kanehisa et al., 2016). All the software and web-based tools were used with their default parameters.

#### 2.5.3 Functional annotation for PGPT (PLaBAse)

The prediction of PGPT associated with the P1 genome (annotated protein sequences) was analyzed using the PGPT-Pred module (with BLASTP+HMMER Aligner/Mapper) of the PLaBase (PLant-associated BActeria web resource) tool v.1.02 (Patz et al., 2021) using default parameters.

#### 2.5.4 Prediction of carbohydrate-active enzymes (CAZymes)

CAZyme-associated genes were annotated in the P1 genome using the dbCAN3 software (Zheng et al., 2023) with DIAMOND, BLAST, and HMMER searches against the CAZy database (Cantarel et al., 2009). The HMMER-based searches were performed using the following thresholds: dbCAN (E-value < 1e−15, coverage > 0.35) and dbCAN-sub (E-value < 1e−15, coverage > 0.35).

#### 2.5.5 Genome mining for AMR genes, associated virulence factors, bacteriocins and secondary metabolite biosynthetic gene clusters

The antibiotic resistance (AMR) genes of P1 were identified using the Comprehensive Antibiotic Resistance Database (CARD) with defined default conditions (Alcock et al., 2023) and the ResFinder tool (Bortolaia et al., 2020). To identify bacterial virulence factors (VFs), the Virulence Factor Database (VFDB) (Liu et al., 2022) was used. The bacteriocin-encoding regions potentially responsible for the antibacterial activity were assessed using the BAGEL4 software (van Heel et al., 2018). The secondary metabolite gene clusters of P1 were identified using antiSMASH 7.0 (Blin et al., 2025).

### 2.6 Statistical analysis

Statistical analyses were performed using GraphPad Prism 8.0. All experiments were conducted in triplicate, and the results are expressed as mean ± standard deviation (SD). For salt and drought stress assays, differences among stress treatments were analyzed using one-way analysis of variance (ANOVA), followed by Dunnett’s multiple comparison test to compare all treatment groups against a single control. Statistical significance was taken at *p* ≤ 0.001.

## 3 Results

### 3.1 Bacterial morphology and identification

**Figure 2:**
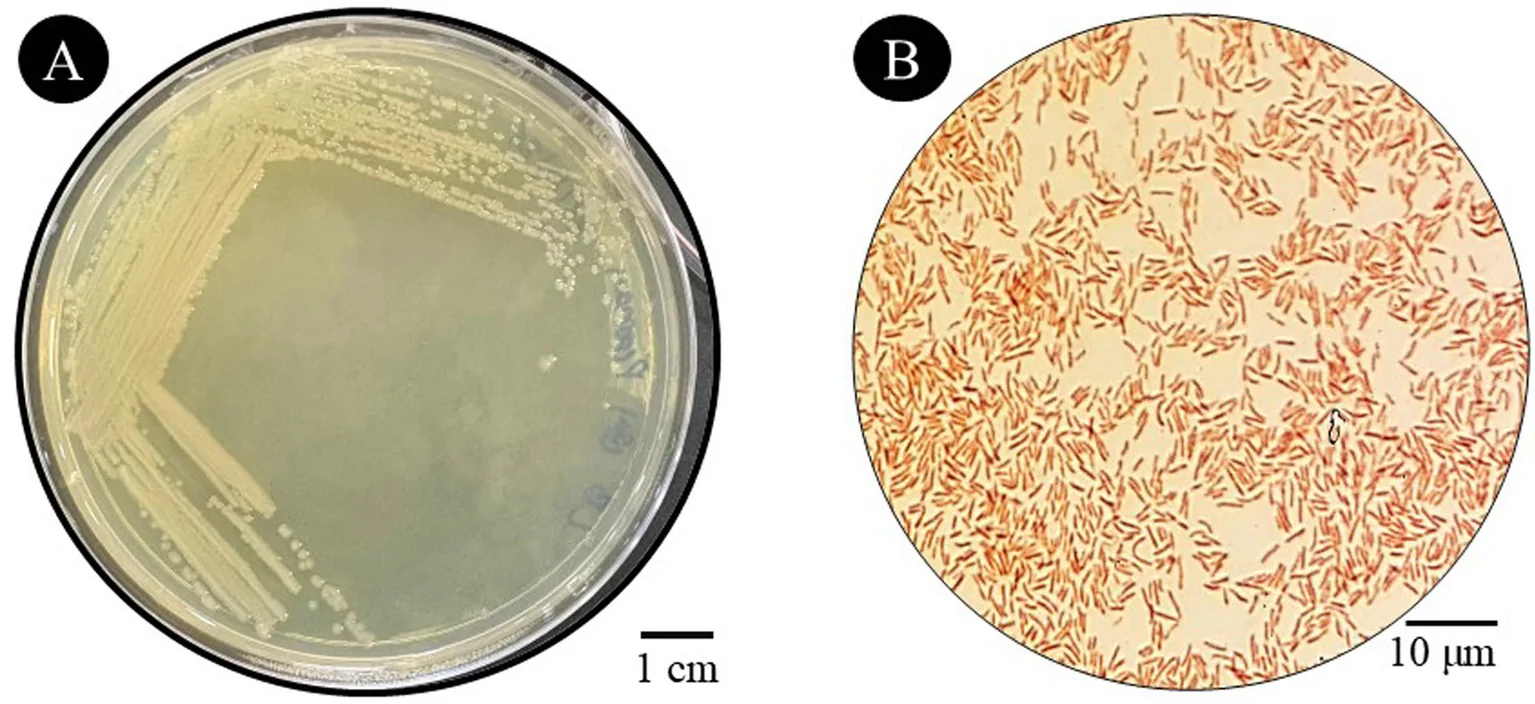
Characterization of *Pseudomonas stutzeri* P1. **(A)** Bacterial endophyte (P1) isolated from an *in vitro* grown potato explant, its growth on LB media, and **(B)** Gram’s staining profile of P1: Scale: 1 cm **(A)** and 10 μm **(B)**.

The isolated bacteria appeared as round and medium-sized colonies on LB medium, with a Gram-negative profile (Figure 2). Based on the MALDI-TOF analysis, the P1 was identified with a score value of 2.04, and classified as *Pseudomonas stutzeri* (a class of plant endophyte) bacteria, reported in potato (Pham et al., 2017).

### 3.2 Biochemical assay-based evaluation of PGPT activity

**Figure 3:**
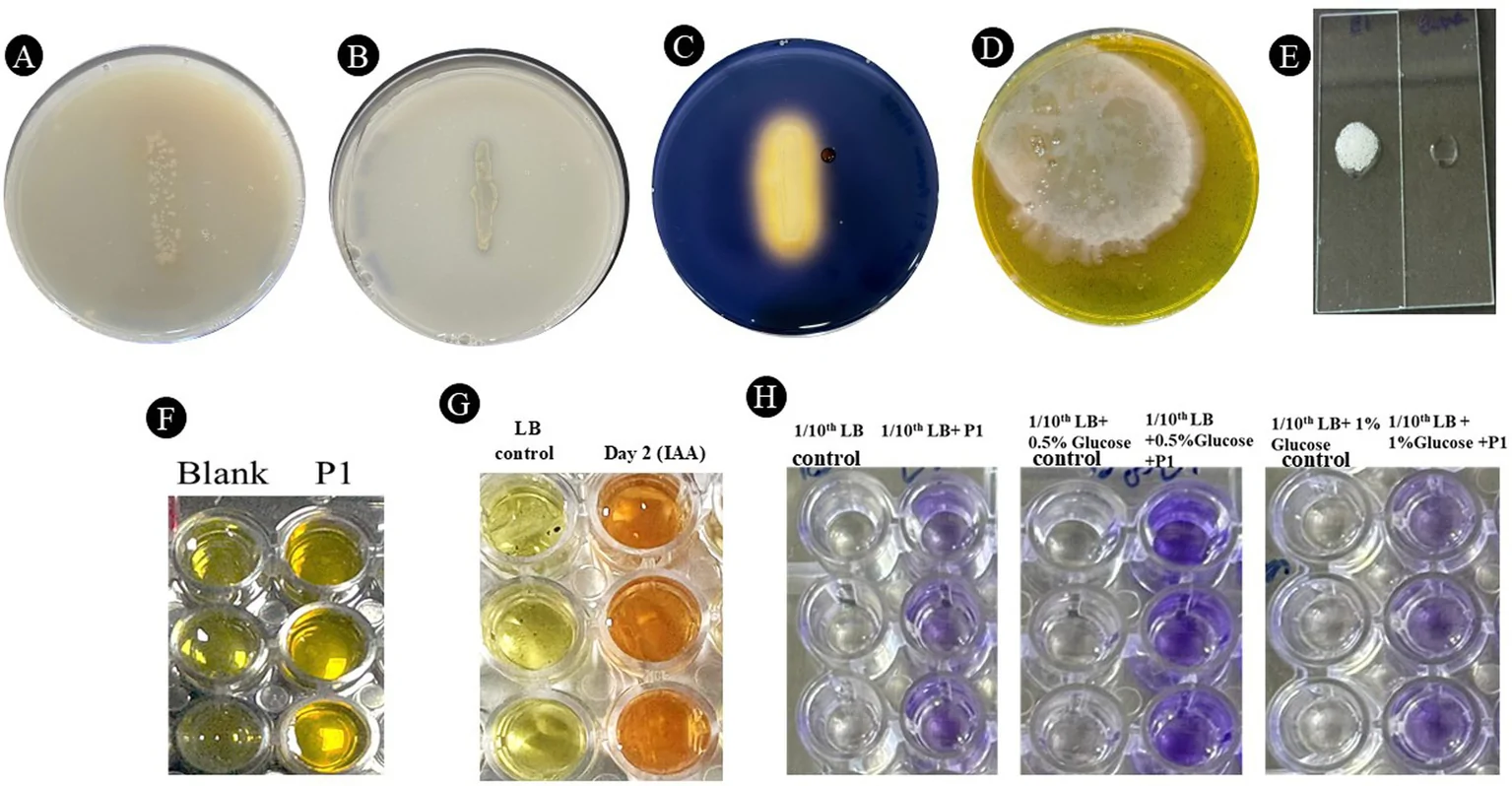
PGPT of *Pseudomonas stutzeri* P1 - qualitative assays: **(A)** Nitrogen fixation ability: Growth of strain P1 on nitrogen-free medium, **(B)** Phosphate solubilization: Formation of a clear halo zone around the colony, **(C)** Amylase production: Starch hydrolysis zone surrounding the bacterial growth after iodine staining, **(D)** Siderophore production: Absence of orange halo formation on CAS agar, **(E)** Catalase production: Bubble formation on addition of H_2_O_2_, and **(F)** Ammonia production: Development of yellow to orange coloration in Nessler’s reagent; **(G)** IAA production: Development of pink to orange coloration, and **(H)** Biofilm formation: Crystal violet staining intensity indicating biofilm formation under different glucose concentrations.

P1 strain was subjected to *in vitro* assays related to traits that help in the promotion of plant growth. The qualitative assays, nitrogen fixation, phosphate solubilization, amylase production, and catalase production showed positive results, whereas siderophore production was not detected (Figures 3A–F). Additionally, phosphate solubilization was measured at 78.14 ± 0.01 μg mL^−1^. The amylolytic activity of *Pseudomonas stutzeri* P1 increased from day 1 to 5. On day 1, the isolate produced about 95.62 mg L^−1^ reducing sugar and had an amylase activity of 2.12 × 10^−4^ U mL^−1^. Meanwhile, on day 5, the reducing sugar concentration increased to 211.17 mg L^−1^, with an amylase activity of 4.69 × 10^−4^ U mL^−1^. The ammonia production of 14.52 μg mL^−1^ was estimated based on the standard ammonium calibration curve. The maximum IAA production was observed on the second day, at 28.37 ± 0.06 μg mL^−1^ (Figure 3G). Furthermore, a strong biofilm production was also observed with BFI of 2.35 (OD > 4 × ODc) for P1 bacterium (Figure 3H) (Table 1).

**Table 1:** PGPT of the P1 strain isolated from a potato plant.

Bacterial strain	Production assay							
	Nitrogen fixation	Phosphate solubilization	Amylase production	Siderophore production	Catalase production	Ammonia production	IAA production	Biofilm production
P1	+ve	+ve	+ve	−ve	+ve	+ve	+ve	+ve
		Phosphate solubilization activity	Amylolytic activity			Ammonia production concentration	IAA production concentration	Biofilm formation index (BFI)
Concentrations		78.14 ± 0.01 μg mL^−1^	4.69 × 10–4 U mL^−1^			14.52 ± 0.09 μg mL^−1^	28.37 *±* 0.06 μg mL^−1^	2.35

### 3.3 Abiotic stress: salt and drought tolerance

**Figure 4:**
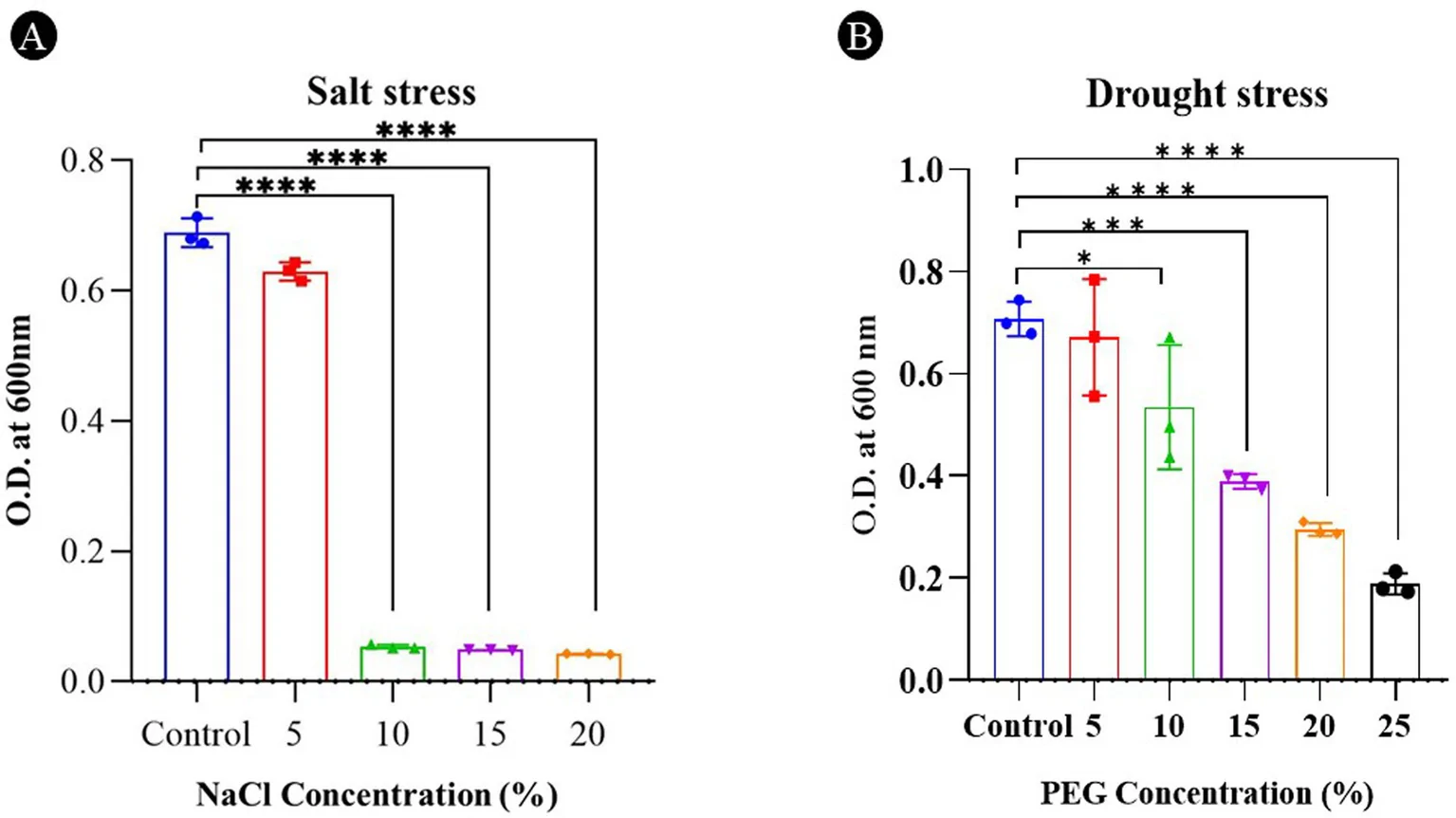
Salinity and drought stress tolerance of *Pseudomonas stutzeri* strain P1. **(A)** P1 strain growth under salinity conditions (5–20% NaCl concentrations) and **(B)** drought conditions (5–20% PEG 4000 concentration). Values are mean ± standard deviation of three replicates. Mean values followed by the triple asterisk mark are significantly different according to Dunnett’s *post hoc* test (***p ≤ 0.001).

The effect of salt (NaCl) and drought (PEG 4000) stress on the P1 strain was assessed. Under salt stress, P1 strain showed a dose-dependent inhibitory effect with survival up to 5% NaCl, while a significant decline in growth was observed at 10–20% NaCl compared to the control (*p* ≤ 0.001; Figure 4A). Similarly, under drought-stress conditions, growth of the P1 isolate decreased in a dose-dependent manner as PEG 4000 concentration increased. The highest growth under stress was observed at 5% PEG 4000, followed by a progressive decline up to 25% PEG 4000, compared to the control (*p* ≤ 0.001, Figure 4B).

### 3.4 Biotic stress- antifungal activity

**Figure 5:**
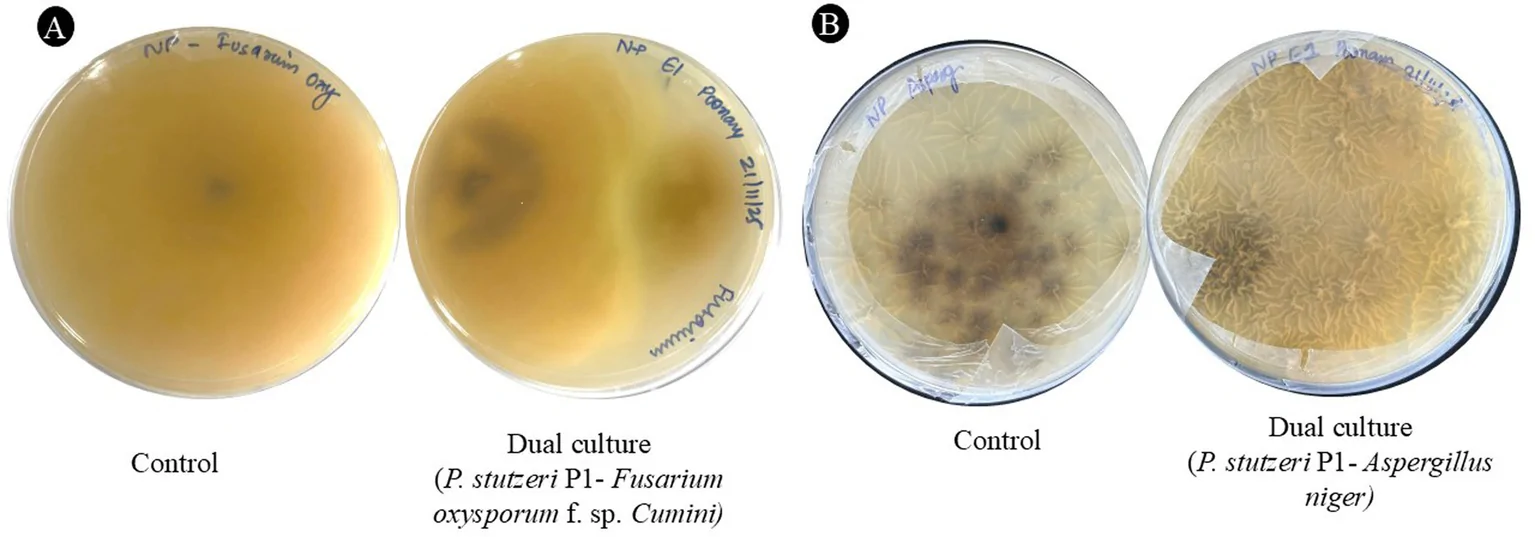
Biocontrol activity of *Pseudomonas stutzeri* strain P1 assessed against **(A)** *Fusarium oxysporum* f. sp. *cumini* and **(B)** *Aspergillus niger*.

The antifungal activity of the P1 strain was assessed with two fungi, *Fusarium oxysporum* f. sp. *cumini* and *Aspergillus niger*. The strain P1 showed potent antifungal activity of 48.9% (control fungal growth-90 mm; treatment fungal growth-46 mm), inhibition of fungal mycelial growth against *Fusarium oxysporum* f. sp. *Cumini* (Figure 5A) under dual culture conditions. Conversely, no antifungal activity was observed against *A. niger* (Figure 5B).

### 3.5 Genome-based taxonomy and assembly statistics

**Figure 6:**
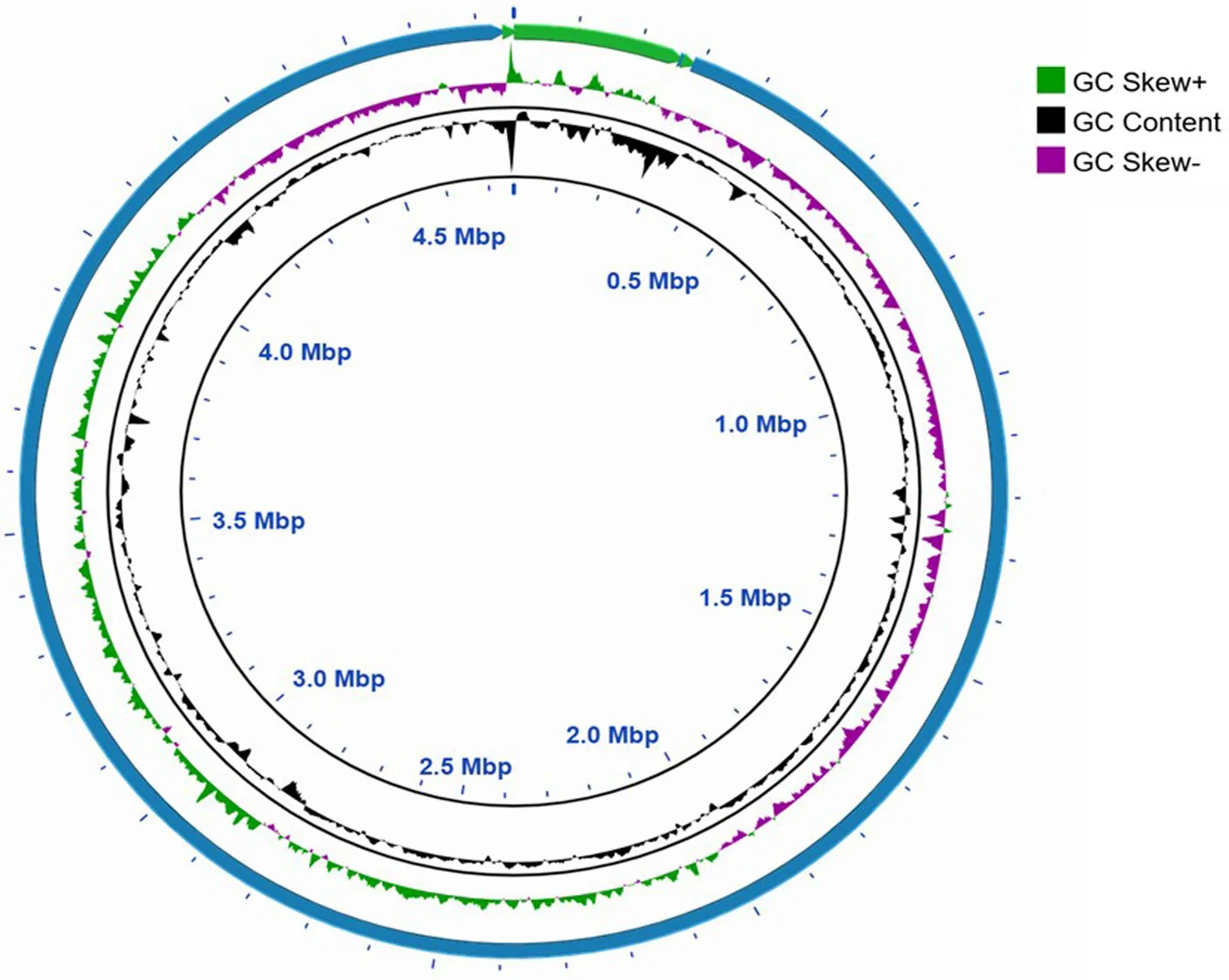
Genomic architecture of the *Pseudomonas stutzeri* strain P1. Circular genome representation of *Pseudomonas stutzeri* P1 generated using Proksee. The genome represents a high-quality draft and open genome consisting of five contigs. The green arrows indicate predicted coding sequences, and the inner circle rings represent positive and negative GC skew profiles in the genome.

Whole-genome sequencing of P1 generated 121.9 MB of raw sequence data and provided a draft genome assembly of approximately 26X coverage. Quality assessment of the Nanopore reads was carried out using FastQC, representing moderate-quality long-read data suitable for draft genome assembly (although per-base quality reduction characteristic of Nanopore sequencing was observed). The assembled genome consisted of 5 contigs with a total genome size of 4.7 Mb and a GC content of 63.5%, indicating a high-quality draft but open genome assembly. QUAST analysis indicated an N50 value (largest contig) of 4,463,208 bp, and BUSCO analysis showed 97.7% completeness with 0% contamination (Table 2). The circular genomic representation generated using Proksee illustrated the arrangement of assembled contigs and GC skew distribution across the genome architecture (Figure 6). The assembled genome and raw sequences are deposited under bio project number PRJNA1432527, Biosample Accession: SAMN56344491, and SRA Accession: SRR38898238.

**Table 2:** Genome assembly statistics and DFAST-based genome annotation features of *Pseudomonas stutzeri* P1.

Features	DFAST	QUAST	BUSCO
Total length (bp)	4,758, 235	4,758, 235	4,758,235
Number of Contigs (sequences)	5	5	5
GC content (%)	63.5	63.54	–
L50	–	1	–
N50	4,463,208	4,463,208	4 Mb
CDS	4,548	–	–
tRNA	66	–	–
rRNA	12	–	–
CRISPR elements	0	–	–
Coding ratio (%)	89.3%.	–	–
Gap ratio (%)	0	–	–
Completeness (%)	-	–	97.7

**Figure 7:**
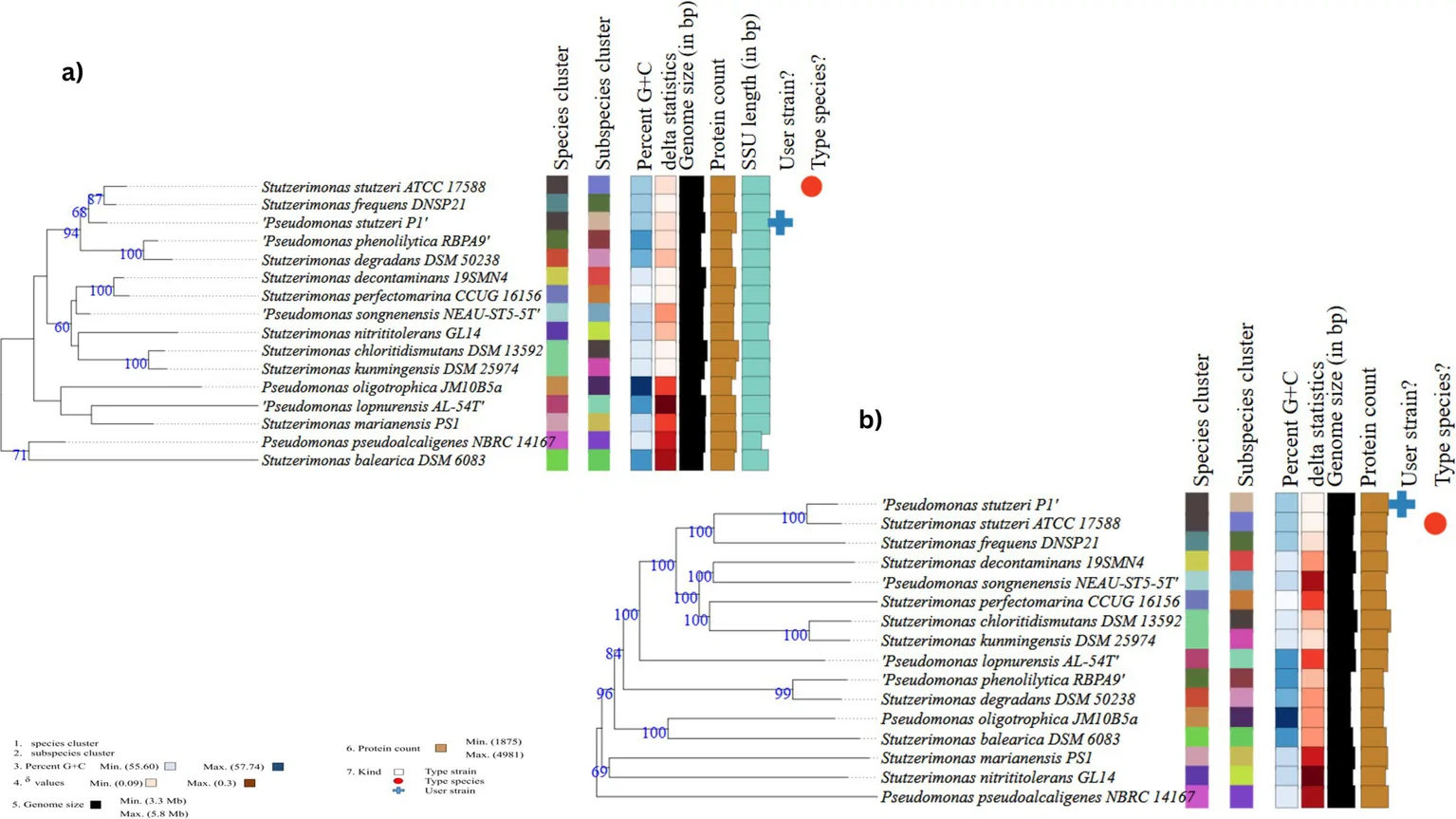
Genome-based identification of *Pseudomonas stutzeri* strain P1 based **(a)** on whole-genome and **(b)** 16S rDNA using the Type Strain Genome Server (TYGS). The tree was estimated using FastME 2.1.456 (Desper and Gascuel, 2006) based on GBDP distances calculated from genome sequences. Branch lengths are scaled in terms of the GBDP distance formula d5; numbers above branches are GBDP pseudo-bootstrap support values from 100 replications. User-provided GenBank accession IDs are shown in parentheses; master record accessions are truncated. Leaf labels are annotated by affiliation to species (1) and subspecies (2) clusters, genomic G + C content (3), *δ* values (4), overall genome sequence length (5), number of proteins (6), and the kind of strain (7). User-provided GenBank accession IDs are shown in parentheses; master record accessions are truncated.

The P1 genome was identified using the PubMLST database, yielding a 100% match to *Pseudomonas stutzeri*. Species-level (Figure 7a) and whole-genome (Figure 7b) confirmation was further supported by TYGS analysis, which revealed a dDDH value of 77.5% with *P. stutzeri* ATCC 17588, exceeding the accepted species delineation threshold of 70%, thereby confirming that strain P1 belongs to the same species. Moreover, the Barnapp tool was used to extract 16S sequences of P1 and its BLAST results, which showed 99.67% sequence similarity to *Pseudomonas stutzeri* ATCC 17588. Furthermore, average nucleotide identity (ANI) was assessed using the DFAST tool (Table 2), which yielded a conclusive match of 98% to P1 (ANI expected values ≥95–96% are generally considered indicative of the same species).

### 3.6 Genome-based functional annotation

**Figure 8:**
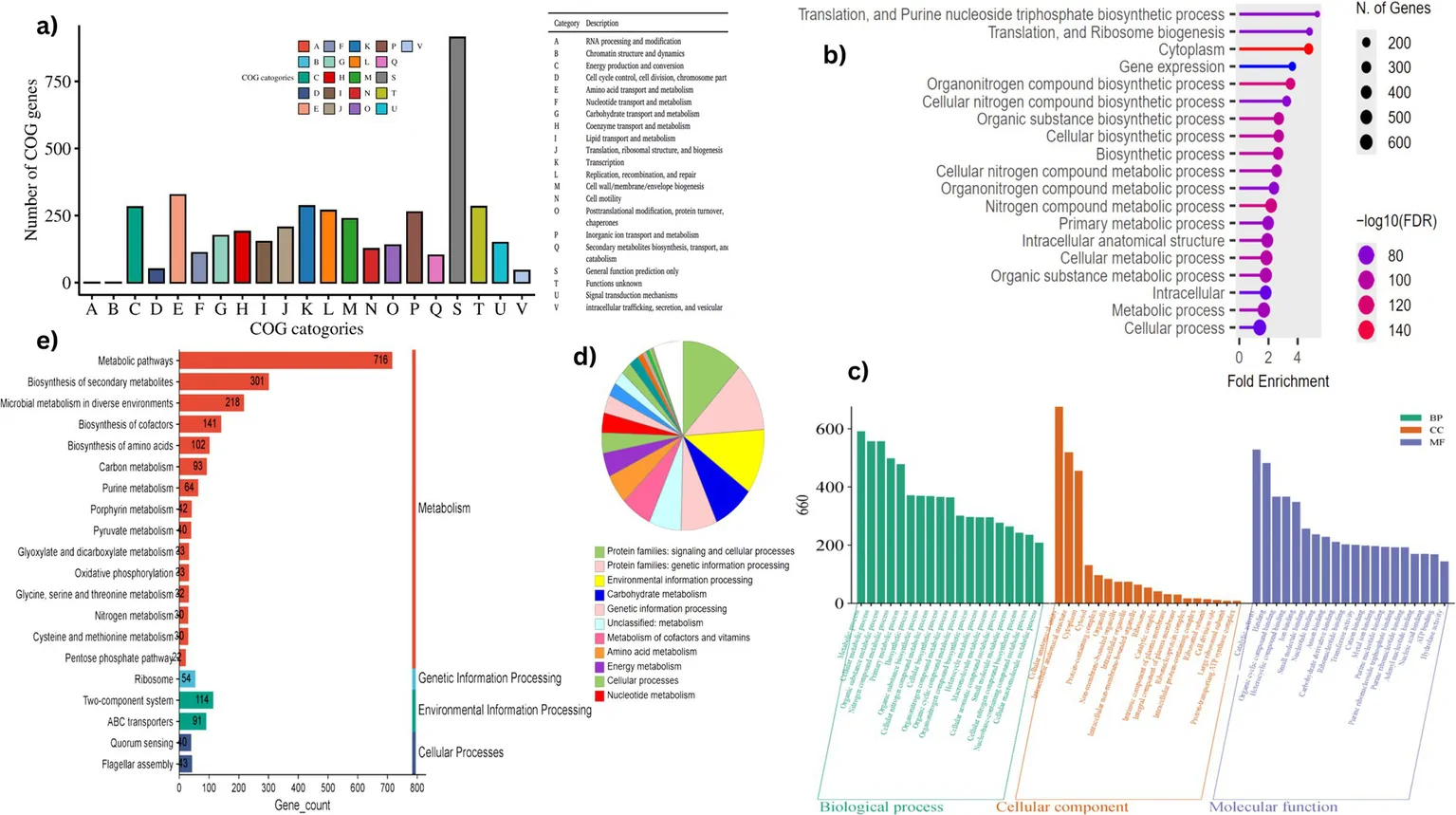
Functional annotation of the *Pseudomonas stutzeri* P1 genome. **(a)** The distribution of genes across various COG functional categories was identified using eggNOG mapper. **(b)** Gene enrichment analysis using ShinyGO analysis representing significantly enriched biological processes and cellular components based on fold enrichment, gene counts, and –log10(FDR) values. **(c)** Gene Ontology (GO) functional classification of the P1 strain, based on the distribution of annotated genes across Biological Process (BP), Cellular Component (CC), and Molecular Function (MF) categories, with bar heights representing the number of genes assigned to each GO term. **(d)** KEGG-based functional annotation representing the distribution of gene families across major KEGG pathways based on gene count, and **(e)** its classification based on protein families.

A total of 4,311 genes (96.9%) were classified into 25 functional clusters of orthologous groups (COGs). The distribution of genes among COG functional categories is presented in Figure 8a, showing that most genes fall in category S, ‘general function prediction only’. Followed by the S category, the E, C, K, L, P, and T categories occupy nearly a similar number of genes. The gene enrichment analysis showed high fold enrichment in translation-related processes such as ribosome biogenesis and purine nucleotide biosynthesis, followed by nitrogen and organic compound biosynthetic/metabolic processes, and cytoplasmic and intracellular components, highlighting dominant core housekeeping and growth-associated functions (Figure 8b). The gene ontology (GO) data (top 21 hits) showed that genes were assigned to biological processes, mainly metabolic and biosynthetic functions. For cellular components, genes were assigned to intracellular and cytoplasmic localization, and molecular functions revealed enrichment of binding and catalytic activities (Figure 8c). Further, KEGG database functional annotation of P1 identified 2,644 genes (59.4%) out of 4,449 entries. These 2,644 genes are distributed across 45 KEGG pathways based on gene counts (Figure 8d), which shows deeper molecular and functional insights. The identified pathways were further grouped into five categories based on the identified protein families: Cellular Processes, Genetic Information Processing, Metabolism, Environmental Information Processing, and Organismal Systems, which were then subdivided into gene-associated subcategories. The data represented a strong prevalence of metabolic pathways with central metabolism and secondary metabolites biosynthesis, followed by the environmental processing system, transport, and cellular processes (Two-component system, ABC transporters, Quorum sensing, and flagellar assembly) (Figure 8e).

### 3.7 Identification of PGPT by PLaBAse

**Figure 9:**
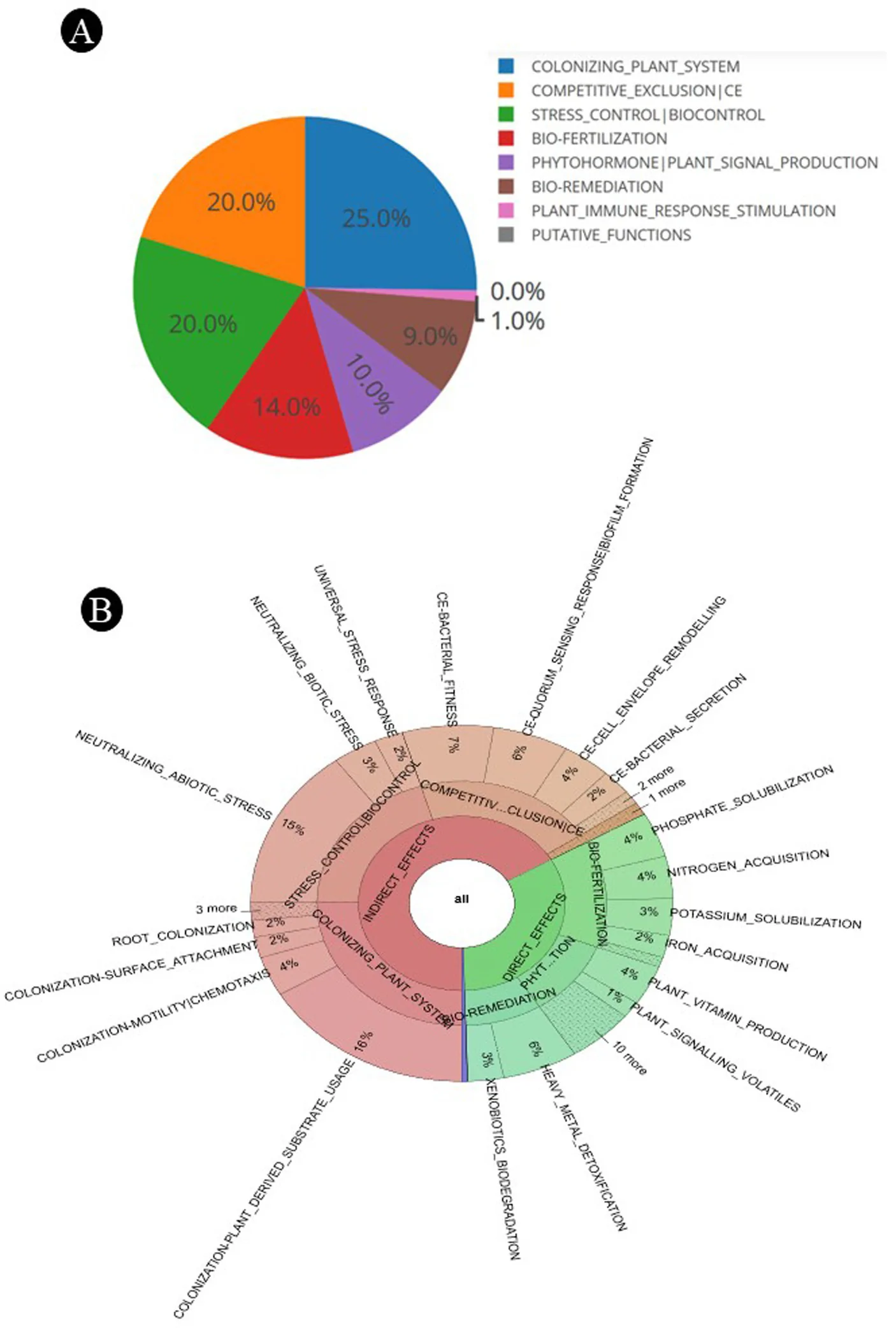
PLaBAse (PGPT-pred tool-based) representation of plant growth–promoting traits (PGPTs) in *Pseudomonas stutzeri* strain P1. **(A)** Overall PGPT-based functional annotation of identified traits. **(B)** The Krona plot representing direct and indirect effects of plant growth-promoting effects. PGPT traits were annotated through BLASTp and HMMER searches against the PGPTBASE, and results are shown at the third level of the six-tier annotation hierarchy.

The PLaBAse database produced a total of 2,574 genes with potential PGPTs in strain P1, which projects direct effects (33.7%) and indirect effects (66.3%) (Figure 9A). The direct effects include biofertilization (14%), phytohormone signal production (10%), and bioremediation processes (9%), whereas indirect effects are related to plant colonization (25%), biocontrol (20%), and competitive exclusion (20%) (Figure 9B). The Krona plot of direct functional traits revealed that PGPTs were predominantly associated with biofertilization, accounting for 52% or 42%, including functions related to nitrogen and iron acquisition, potassium solubilization, general nutrient solubilization, and cation mineralization. Phytohormone production constituted 30% or 23% of the traits, largely represented by IAA biosynthesis and other hormone-related pathways. The remaining 25% of PGPT were associated with bioremediation, encompassing activities such as heavy-metal detoxification and xenobiotic degradation (Supplementary Figure S1).

### 3.8 Gene mining of PGPT of strain P1

Genetic insights into putative genes associated with PGPT were elucidated for the P1 strain. The data showed the presence of gene clusters associated with nitrogen metabolism and fixation, phosphate solubilization, biofilm formation, root colonization and phytohormone production, confirming the PGPT potential. The key genes involved in nitrogen fixation and metabolism (*nar, nos, gln, npd*, and *nrt*) and phosphate solubilization (*pho* and *pst*) promote optimal nutrient absorption. Genes encoding biofilm formation, chemotaxis, and motility (*flg, mot, aer*, and *xer*) demonstrate effective root colonization activity. Additionally, genes responsible for phytohormone production, such as auxin (IAA) and cytokinin, and genes involved in terpenoid biosynthesis pathways, strengthen plant growth-related traits (Table 3).

**Table 3:** Putative genes involved in various PGPT in the *Pseudomonas stutzeri* P1 genome.

PGPT	Gene names and KO IDs	References	P1 genome
Nitrogen Metabolism, Nitrogen cycle, Nitrogen fixation	*narG, narZ, nxrA* (K00370), *narH, narY, nxrB* (K00371), *narI, narV* (K00374), *nosZ* (K00376), *norC* (K02305), *norB* (K04561), *gltB* (K00265), *glnA, GLUL* (K01915), *narI, narV* (K00374), *nosZ* (K00376), *ncd2, npd* (K00459), *NRT2, narK, nrtP, nasA* (K02575)	Patel et al. (2025a,b)	+
Phosphate solubilization	*phoB* (K07657), *phoH* (K06217), *phoR* (K07636)	Kang et al. (2020)	+
	*pstA* (K02038), *pstB* (K02036)	Patel et al. (2025a,b)	+
	*pstQ* (K07637)*, pstP* (K08484), *phoP* (K07658), *OmpR* (K07659)	−	+
Biofilm formation	*fliC* (K02406), *pelf, mdcF* (K13936), *aoxS, pvdQ* (K07116)	−	+
Flagellar Biosynthesis	*flgB*, (K02387), *flgC* (K02388), flg*D* (K02389), *flgE* (K02390), *flgF* (K02391), *flgG* (K02392), *flgH* (K02393), *flgI* (K02394)	Singh et al. (2021)	+
	*flgJ* (K02395), f*lgL* (K02397), *flgM* (K02398), *flgN* (K02399), *flgA* (K02386)	−	+
Root colonisation	*motA* (K02556)*, motB* (K02557) *minC* (*K03610*), *minD* (K03609) *xerD* (K04763), *xerC* (K03733)	Singh et al. (2021)	+
	*aer* (K03776)	Patel et al. (2025a,b)	+
Peroxidase	*OsmE* (K04064), *mcp* (K03406)	Gupta et al. (2014)	+
IAA production	*trpD* (K00766), *trpC* (K01609), *trpA* (K01695), *trpB* (K01696)	Patel et al. (2025a,b)	+
Cytokinin biosynthesis	*miaA* (K00791), *miaB* (K00791) *miaE* (K06169), *log*	Patel et al. (2025a,b)	+
Terpenoid biosynthesis	*dxs* (K01662), *dxr* (K00099), ispD (K00806), *ispE* (K00919), *ispF* (K00991), *ispG* (K01662), *ispH* (K01770), idi (K01823), *ispA* (K02523), *ggpS* (K03186), *uppS* (K03526), *uppS-like* (K03527), *ispS-like* (K13789)	Chen et al. (2021)	+
Carotenoid biosynthesis	*crtB* (K0229), *crtI* (K10027), *crtY* (K06443), *crtX* (K14596), *crtZ* (K15746)	Sandmann (2023)	+

The data also elucidated genes conferring stress tolerance by the presence of heat shock proteins (*htpX, hsp33, hsp15*), oxidative stress (*trxAB, dsbD, SOD1/2*), heavy metal stress (*cusR*/*S*), benzoate degradation (*benABCKE_xylX* operon*, catABC*), salinity (*ectABCD* operon), and drought (*yciG*) stress, which highlight multi-stress responsive characteristics of the P1 genome (Table 4).

**Table 4:** Putative genes involved in stress responsiveness in the *Pseudomonas stutzeri* P1 genome.

Stress-related proteins	Gene names and KO IDs	References	P1 genome
Heat shock proteins	*htpX* (K03799), h*slJ* (K03668)	−	+
	*hsp33* (K04083), *hsp15* (K04762), *hsp20* (K13993)	Patel et al. (2025a,b)	+
Oxidative stress	*trxB* (K00384), *trxA* (K03671), *dsbD* (K04084), SOD2 (K04564), SOD1 (K04565)	Patel et al. (2025a,b)	+
Heavy metal stress	*czcD* (K16264), cusS (K07644), *cusR* (K07665)	Patel et al. (2025a,b)	+
Benzoate degradation	*benA_xylX* (K05549), *benB_xylX* (K05550), *benD_xylX* (K05783), *benC_xylZ* (K05784)*, benK* (K05548), *benE* (K05782) *catA* (K03381), *catB* (K01857), *catC* (K03464), *pcaD* (K01055)	Xiang et al. (2020)	+
Salt stress	*ectB* (K00836)*, ectA* (K06718)*, ectC* (K06720)*, ectD* (K10674), *rnfA* (K00350), *rnfC* (K03615), *rnfG* (K03612), *rnfE* (K00349), *rnfD* (K00347)	−	+
Drought stress	*yciG* (K06884), *erfK* (K16291), SOD1 (K04565), *CAT1* (K18118), *gpx* (K00432)	−	+

### 3.9 Characterization of other traits in P1 genome

#### 3.9.1 CAZymes

To identify CAZyme-associated genes in the P1 genome, three different complementary annotation approaches were used, which include HMMER, dbCAN, and DIAMOND. The P1 genome revealed a diverse repertoire of carbohydrate-active enzymes, including auxiliary activities (AAs), glycoside hydrolases (GHs), glycosyltransferases (GTs), carbohydrate esterases (CEs), carbohydrate-binding modules (CBMs), and polysaccharide lyases (PLs). Among these, glycoside hydrolases constituted the most abundant CAZyme class, represented by multiple families, included family GH2, GH3, GH8, GH13, GH15, GH17, GH18, GH19, GH20, GH23, GH31, GH37, GH39, GH43, GH50, GH67, GH73, GH77, GH92, GH97, GH102, GH103, GH104, GH109, GH144, GH153, and GH166. This was followed by glycosyltransferase families such as GT1, GT2, GT4, GT5, GT9, GT19, GT20, GT28, GT30, GT35, GT41, GT51, GT60, GT83, and GT104. Additionally, several carbohydrate-binding modules (CBM12, CBM22, CBM32, CBM48, CBM50, CBM51, CBM73, and CBM91) and carbohydrate esterase families (CE1, CE4, CE9, CE11, CE12, and CE20) were identified. Auxiliary activity enzymes (AA1, AA3, AA4, AA6, AA10, and AA12) and polysaccharide lyase families (PL5, PL6, PL7, PL12, and PL17) were also present in comparatively lower abundance.

#### 3.9.2 Antimicrobial resistance genes

The CARD analysis identified resistance-associated genes showing 100% sequence similarity to determinants conferring resistance to aminoglycosides, fluoroquinolones, carbapenems, cephalosporins, penicillins, and other *β*-lactam antibiotics. Furthermore, genes exhibiting 98% similarity were associated with resistance to fluoroquinolones, diaminopyrimidines, and phenicols. However, analysis using the ResFinder tool did not detect any acquired antimicrobial resistance genes in the P1 genome, indicating the absence of clinically relevant resistance determinants.

#### 3.9.3 Virulence- associated factors

The virulence factor database was used to assess adherence, antiphagocytosis, iron uptake, quorum sensing, regulation, and other virulence-associated genes from the P1 genome. The results showed that adherence-related flagella factors were predominant, followed by pili biosynthesis and subsequently alginate regulation (Table 5).

**Table 5:** The number of virulence factors associated with the P1 genome was identified using the virulence factor database.

VF class	Virulence factors	Genes
Adherence	Flagella	46
	LPS O-antigen (*P. aeruginosa*)	1
	Type IV pili biosynthesis	16
	Type IV pili twitching motility-related proteins	8
Antiphagocytosis	Alginate biosynthesis	4
	Alginate regulation	11
	Capsular polysaccharide (Vibrio)	1
	Capsule (Klebsiella)	1
Iron uptake	Pyoverdine receptors	3
Quorum sensing	Acylhomoserine lactone synthase	1
Regulation	GacS/GacA two-component system	2
Others	O-antigen (Yersinia)	1

#### 3.9.4 Bacteriocins- related gene clusters and secondary metabolites

The complete genome sequence of P1 was analyzed to identify putative bacteriocin gene clusters within secondary metabolite biosynthesis gene clusters, as well as ribosomally synthesized and post-translationally modified peptides. As a result, BAGEL 4.0 cannot detect any bacteriocin clusters in the P1 genome. The antiSMASH 7.0 indicated the presence of betalactone, NRPS-like compounds, ectoine, terpenes, NI-siderophores, terpene precursors, NAGGN, RiPP-like, and redox cofactors, including betalactone. *Corynecin* I, II, and III showed low confidence in similarity; desferrioxamine E showed medium confidence in similarity, whereas ectoine and carotenoid displayed high confidence in similarity.

## 4 Discussion

The current research presents an integrated genomic and functional characterization of the endophytic bacterium *Pseudomonas stutzeri* strain P1, which demonstrates its strong potential as a plant growth–promoting bacterium (PGPB) in potato. The collective assessment of whole-genome sequencing, functional annotation, and biochemical assays revealed that strain P1 possesses several traits related to plant growth promotion, stress tolerance, and biocontrol activity. These findings indicate that strain P1 is well adapted to plant-associated environments and may contribute to sustainable crop production.

The taxonomic identification of *P. stutzeri* P1 strain using PUBMLST revealed 99.67% sequence similarity to *Pseudomonas stutzeri* ATCC 17588, whereas ANI analysis showed 98% genome similarity. Furthermore, TYGS confirmed the taxonomy of strain P1 with a dDDH value of 77.5% relative to *P. stutzeri* ATCC 17588, which is reportedly known for its stimulatory effects on PGPT (Jiang et al., 2022; Gao et al., 2024). The sequencing results for genome assembly indicated a high-quality genome comprising five contigs, totaling approximately 4.75 Mb, with a coding density of 89.3% and a completeness of 99.7%, as assessed by BUSCO. Similarly, QUAST data indicate high quality, with a limited number of contigs, indicating low fragmentation and near-complete genome assembly (Gurevich et al., 2013). These results indicate that the P1 genome represents a near-complete assembly with minimal fragmentation and genome characteristics (4.75 Mb, 63.5% GC content), which are comparable to those of *Pseudomonas stutzeri* strains isolated from rice, soybean, and poplar ecosystems, whose genomes typically range between ~4.5–5.2 Mb with GC content approximately 60–65% (Annapurna et al., 2017; Pérez-Padilla et al., 2025; Deng et al., 2023).

Previous studies have reported that *P. stutzeri* showed broad environmental adaptability (Chauhan et al., 2023), stress tolerance and biocontrol potential (Yang et al., 2025), as well as effective plant colonization ability mediated through biofilm formation and motility mechanisms (Höflich et al., 1995; Preston, 2004; Silby et al., 2011). Consistent with these reports, biochemical assays in the present study confirm various PGPTs estimated through the genome analysis. Strain P1 tested positive for various PGPT traits like nitrogen fixation, phosphate solubilization, catalase production, amylase activity, ammonia production, and IAA production. The strain also represented strong biofilm-forming ability, indicating effective colonization potential. Furthermore, stress tolerance assays demonstrated that strain P1 can grow under salinity and drought stress conditions. Furthermore, biocontrol assays showed that the P1 strain exhibited inhibitory activity against *Fusarium oxysporum* f. sp. *cumini*, indicating antifungal potential. Altogether, these biochemical observations strongly corroborated the genomic predictions and demonstrated the functional expression of plant-beneficial traits in P1.

Functional annotation of the P1 genome revealed broad metabolic versatility and environmental adaptability, features commonly reported in plant-associated endophytic bacteria. The COG annotation, representing the number of genes allocated to COG categories (96.9%), shows a predominance of categories with general function prediction only (S), followed by core cellular processes (K) and replication and repair (L), underscoring the environmental adaptability and genetic stability. Similar distributions of COG categories have been found associated with plant-associated bacterial genomes showing ecological fitness and stress adaptation (Deng et al., 2023). Moreover, ShinyGO enrichment analysis showed dominance of translation-related and nucleotide biosynthesis processes, highlighting active growth and biological functions. The GO analysis further supported these data by indicating enrichment in metabolic and biosynthetic biological processes, as well as cytoplasmic localization and molecular functions. The KEGG-based analysis encompassed broad classifications of metabolic pathways, genetic functions, cellular processes, and environmental responses. The *in-depth* genome annotation of the P1 strain, from the perspective of plant-associated growth traits, identified multiple plant-associated metabolic pathways and their corresponding genes involved in plant growth promotion, stress tolerance, and biocontrol activities through joint analysis of KEGG, PGPT-pred, and biochemical assays. The data corroborate the plant endophyte genome, which covers 57.0 to 60.8% of annotated genes in KEGG orthology (Mažeikiene et al., 2025). The PGPT-Pred analysis of the P1 genome further classifies proteins by function into direct (33.7%) and indirect (66.3%) effects on PGPT. Similar studies have reported direct and indirect effects of plant endophytes on plants (Maddock et al., 2022; Ficca et al., 2025; Patel et al., 2025a).

The comprehensive analysis of the P1 genome in this study showed the presence of different genes associated with PGPT and phytohormone production (Table 3). Similarly, various studies on plant endophytes such as *Pseudomonas granadensis* CT364 (Torrescassana et al., 2025), *Pseudomonas* sp. (Thakur et al., 2023), *Pseudomonas* sp. En3 (Deng et al., 2023) have also reported the occurrence of genes associated with PGPT and phytohormone production. Genome analysis of P1 revealed the presence of genes associated with nitrogen metabolism and denitrification pathways, which correlated with the experimentally observed growth of strain P1 on nitrogen-free media, confirming its nitrogen-fixing potential. A cluster of gene sets was identified in the P1 genome involved in biological nitrogen fixation, metabolism, and the nitrogen cycle, such as *nar, nrt, nor, nxr, glt,* and *nos* (Table 3; Supplementary Figures S2A,B). The data were further supported by the growth of the P1 strain on nitrogen-free media and ammonia production up to 14 μg mL^-1,^ and findings from earlier research, which confer *Pseudomonas* with nitrogen-fixing ability (Pham et al., 2017; Deng et al., 2023).

Phosphate solubilization activity was experimentally validated through halo zone formation on Pikovskaya agar plates, as well as quantitative phosphate solubilization in NBRIP medium, which was measured as 78.14 ± 0.01 μg mL^-1,^ and the data was supported by the presence of *pho* and *pst* gene clusters involved in phosphate metabolism and transport, wherein, PhoB/PhoR (two-component regulatory system) acts as a sensor of environmental phosphorus availability, while *pst*AB transporters participate in the uptake of phosphorus into the cell (Deng et al., 2023). Similar studies represent *Pseudomonas* sp., positive for phosphate solubilizing activity (Bargaz et al., 2021; Bakki et al., 2024).

Phytohormone production represents another important mechanism of plant growth promotion. A set of key regulatory genes related to tryptophan metabolism was identified, which are highly correlated to bacterial IAA synthesis involved in plant growth and in mitigating abiotic stresses (Etesami and Glick, 2024). A *trp*ABCD cluster was identified in the P1 genome. The present findings align with similar results of other bacterial genomes producing IAA hormone and genes associated with tryptophan metabolism, such as *Pseudomonas aeruginosa* B-18 (Singh et al., 2021), *Bacillus halotolerans* Q2H2 (Wang et al., 2024), *Brucella* sp. PM1 (Patel et al., 2025a). Supporting the data, the P1 strain produced IAA up to 28.37 ± 0.06 μg mL^−1^ in 1% tryptophan. Similarly, the genes corresponding to cytokinin production, *mia*ABE, and *log* [*cytokinin-producing enzyme, “Lonely guy,”* (Akhtar et al., 2020)] were also identified, corroborating data with endophyte P1 (Patel et al., 2025a); and in *Priestia aryabhattai* and *Paenibacillus* sp., cytokinins were associated with improving chlorophyll synthesis and delaying plant senescence (Almirón et al., 2025).

In the genome data for P1, many groups of genes were identified encoding terpenoid biosynthesis (*ispADEFGH, dxs, dxr, uppS*, and *ggpS*) (Supplementary Figure S3) and carotenoid biosynthesis (*crtBIYXZ*). The studies showed that endophytic bacteria encoding terpenoid biosynthetic genes could boost the terpenoid accumulation in the host system and/ or play a significant role in biotic or abiotic stress regulation (Chen et al., 2021). Such ability of endophytic microorganisms to synthesize terpenoid compounds resembling those of their host plants has attracted significant commercial interest (Jayaram et al., 2021). Whereas carotenoids protect against oxidative stress and support plant growth (Ram et al., 2020).

Root colonization is the initial step in establishing an interaction between the plant and bacterium. The P1 genome represents a whole set of machinery involved in flagellar biosynthesis (*flgABCDEFGHIJKLMN*) (Supplementary Figure S4A), root colonization (*motABCD, xerCD, aer*) (Supplementary Figure S4B), and biofilm formation (*fliC, pelf, mdcF, aoxS, pvdQ*), showing a strong interaction at the primary level. The data is also supported by biochemical tests showing a strong positive biofilm formation index of 2.35 with 0.5% glucose. The data align with genes present in other endophytic bacteria with good root colonization (Adeleke et al., 2021; Patel et al., 2025a).

The study also clustered various gene sets associated with multiple biotic and abiotic stresses, including heat shock, cold shock, oxidative stress, and heavy metal stress. Additionally, genes were identified for tolerance against salt stress (*ectABCD and rnfACEDG*), drought stress (*yciG*, *erfK*, *SOD1, CAT1*, *gpx*), and chemical stress (benzoate degradation) (*benABCDEKX _xylX*, *catABC, pcaD*) (Supplementary Figure S5). The data were supported by a biochemical assay with the growth of the P1 strain in salt and drought stress conditions. Additionally, the P1 genome showed biotic stress tolerance against fungi (*Fusarium oxysporum* f. sp. *cumini*). The data align with reports of *P. stutzeri* PSIISS-1 against *F. oxysporum* var. *ciceri* (Kumar et al., 2022), *Pseudomonas stutzeri* ISE12 against salt stress (Szymańska et al., 2022; Dehnavi et al., 2024), *Pseudomonas* spp. against drought stress (Sandhya et al., 2010; Yasmin et al., 2022; Yan et al., 2025), and *Pseudomonas chlororaphis* showed benzoate degradation (Esikova et al., 2021).

The CAZyme repertoire of *Pseudomonas stutzeri* constitutes a specialized enzymatic toolkit that supports its role as a potato growth-promoting endophyte (Singh et al., 2022; Alshareef, 2024). The dominant glycoside of hydrolase families (GH2, GH3, GH13, GH43, GH73, and related groups) enables efficient utilization of plant-derived polysaccharides and root exudates, facilitating nutrient acquisition and endophytic colonization (Boutard et al., 2014; Tashkandi and Baz, 2023). Notably, GH43 and GH30 may act as microbe-associated molecular patterns, eliciting induced systemic resistance in potato plants (Yu et al., 2024). Collectively, these enzymes promote bacterial survival within the host while contributing to plant nutrition and enhanced defense against pathogens (Rutkowska et al., 2025). Glycosyltransferases (GT1, GT2, GT4, GT28, GT35) support exopolysaccharide biosynthesis, facilitating stable biofilm formation and effective root colonization (Maiti et al., 2025). The carbohydrate-binding modules (CBM12, CBM22, CBM32, CBM48, CBM50) enhance enzyme–substrate interactions, improving polysaccharide degradation efficiency, while carbohydrate esterases (CE1, CE4, CE9, CE11, CE12) broaden substrate utilization through deacetylation of plant polymers (Yuan et al., 2022; Krzyżanowska et al., 2023; Saati-Santamaría et al., 2025). Auxiliary activity enzymes (AA1, AA3, AA4, AA6, AA10, AA12) contribute to oxidative processes involved in nutrient cycling and lignocellulose degradation (Udaondo et al., 2018; Alshareef, 2024). In parallel, polysaccharide lyases (PL5, PL6, PL7, PL12, and PL17) act synergistically with glycoside hydrolases to degrade pectic and acidic polysaccharides, potentially releasing bound phosphate and micronutrients and supporting the phosphate-solubilizing capacity of plant growth-promoting *Pseudomonas* strains (Sah et al., 2021; Mehmood et al., 2023).

The identification of a broad range of virulence-associated genes in the P1 genome was associated with adhesion-related factors, likely playing a central role in the early stages of host colonization (Dubey et al., 2022). The dominance of flagellar genes is consistent with their dual function in motility and surface attachment, as well as their involvement in regulating other virulence-associated pathways (Haiko and Westerlund-Wikström, 2013). Similarly, the abundance of pili biosynthesis genes aligns with their well-documented role as primary adhesins that facilitate strong host attachment, biofilm development, and enhanced persistence through immune evasion (Chen et al., 2020; Cangui-Panchi et al., 2023). Moreover, the presence of partial alginate regulatory genes suggests the potential for mucoid biofilm formation (Mahajan et al., 2021). Overall, most classical virulence factors, such as T3SS/T6SS, toxins, phytotoxins, proteases, and phenazines, are completely absent, suggesting that *P. stutzeri* is a non-pathogenic strain, with strong motility and colonization ability.

**Figure 10:**
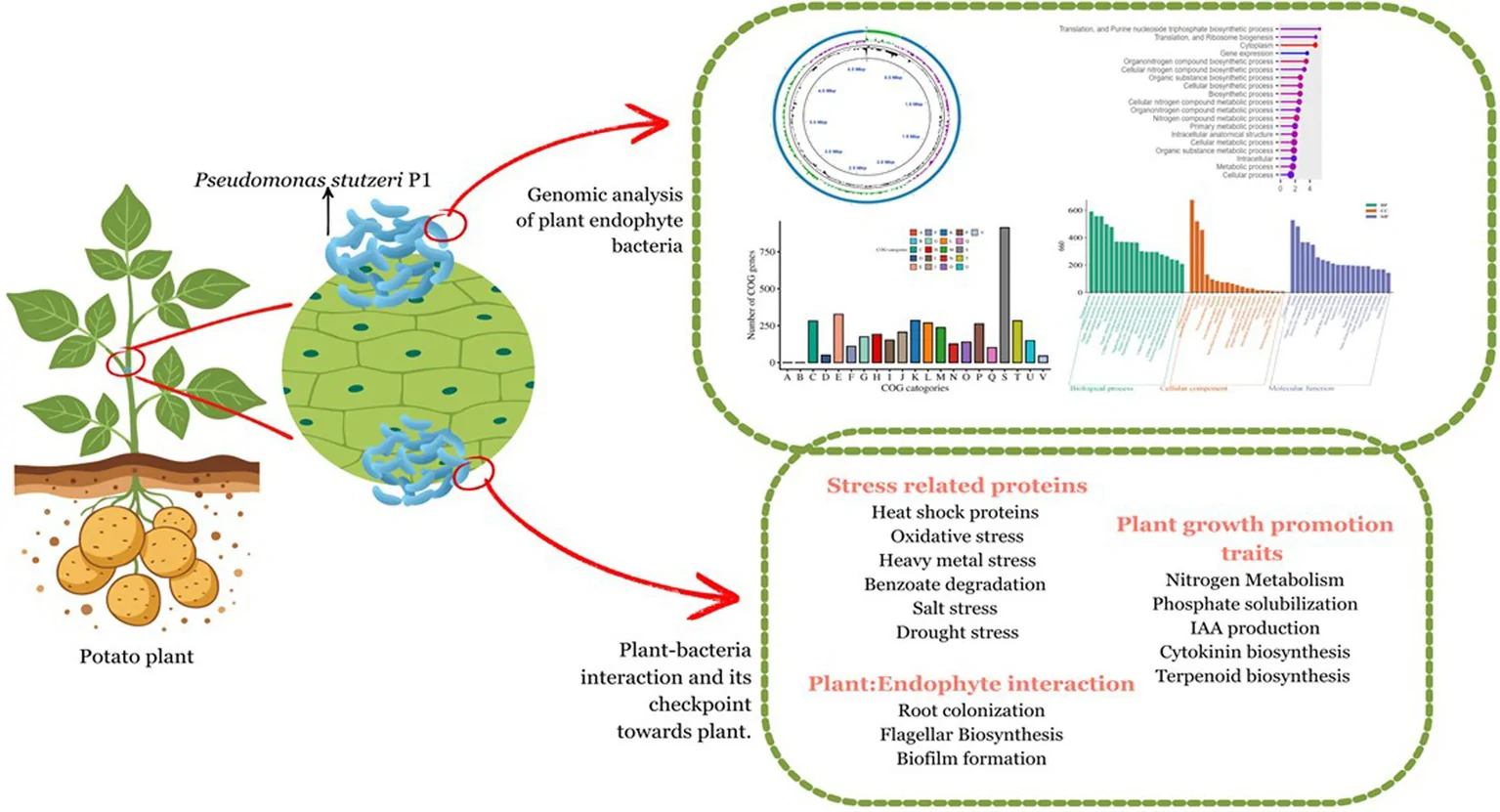
Whole-genome sequencing of the potato endophyte *Pseudomonas stutzeri* P1 revealed a diverse repertoire of genes associated with plant growth promotion, plant–microbe interactions, and stress tolerance, highlighting its potential as a beneficial bioinoculant for sustainable crop production.

Overall, the study on whole-genome sequencing of the potato endophyte *Pseudomonas stutzeri* P1 revealed a diverse repertoire of genes associated with plant growth promotion, plant–microbe interactions, and stress tolerance, highlighting its potential as a beneficial bioinoculant for sustainable crop production (Figure 10, original work, prepared in Canva).

## 5 Conclusion

The isolated strain P1 was identified as *Pseudomonas stutzeri*, a plant-associated endophyte with strong plant growth–promoting potential. Biochemical assays confirmed with multiple PGPT, including nitrogen fixation, phosphate solubilization, IAA production, biofilm formation, root colonization, and tolerance against abiotic (salt and drought stress). The strain P1 also exhibited biocontrol activity against *Fusarium oxysporum* f. sp. *cumini* (biotic stress). Whole-genome sequencing revealed a high-quality, complete genome, whereas functional annotation data showed enrichment with genes involved in metabolism, environmental adaptation, and plant–microbe interactions. Comprehensive gene mining and PGPT-pred analysis further validated the presence of direct and indirect PGPT-associated genes. The analysis of the P1 genome for other traits indicates that the richness of the CAZyme repertoire supports efficient utilization of plant-derived carbohydrates and, in turn, promotes nutrient uptake and enhanced plant defense. The predominance of genes associated with adhesion and motility suggests effective root colonization, and the absence of major pathogenicity-associated factors (toxins, secretion system, phytotoxins, antimicrobials) confirms its non-pathogenic nature. Overall, these features highlight the potential of P1 strain for application in sustainable agriculture and stress-resilient crop improvement.

## Data Availability

The original contributions presented in the study are publicly available. This data can be found here: PRJNA1432527, Biosample Accession: SAMN56344491 and SRA Accession: 597 SRR38898238.

## Footnotes

1
https://proksee.ca/
